# Illegal markets: An important domain for applied science

**DOI:** 10.1073/pnas.2513028123

**Published:** 2026-08-10

**Authors:** Jonathan P. Caulkins, Beau Kilmer, Peter Reuter

**Affiliations:** ^a^ Carnegie Mellon University, Heinz College, Pittsburgh, PA 15213; ^b^ Carnegie Mellon University, RAND Drug Policy Research Center, Pittsburgh, PA 15213; ^c^ University of Maryland School of Public Policy, College Park, MD 20742

## 1. Motivation for Studying Illegal Markets

This Special Feature on illegal markets gathers, perhaps for the first time, papers by some of the world’s leading experts on nine different illegal markets. All are tasked with describing “their” market via a common analytic lens that focuses on the markets’ structure and how they operate. Two additional papers synthesize the main findings from these articles, focusing on analytic commonalities and implications for policy. Collectively, these 11 papers raise questions that may motivate other researchers to work on these topics, possibly spurring conversations about whether illegal markets should become a more prominent field of scientific inquiry.

Illegal markets are certainly a large enough domain of human activity to warrant scholarly attention. By the nature of their illegality, high quality estimates are scarce, but at least two markets likely generate more than $250 billion in annual sales (drugs and counterfeit goods); at least one other generates more than $100 billion annually (commercial sex). Thus, it seems plausible that illegal markets collectively could generate total revenues on the order of $1 trillion per year worldwide—which would put them on par with annual revenues of the global consumer electronics industry (1).

One premise of this special issue is that illegal markets and the supply chains that support them are a worthy topic of scientific inquiry. That turns out to be a novel claim. Illegal markets have simply not been a major topic of interest to economists, the discipline that would most plausibly study them. For example, Nobel laureate Alvin Roth, in his magisterial review of markets and market design, describes illegal markets but offers no specific analytic insights about them (2). Gary Becker, one of the giants of microeconomics, wrote occasionally about drug markets but forced on them the presumption that they were analytically no different than legal markets (e.g., ref. 3).

Economists have also not contributed much empirically. Lacking good quantitative data (e.g., on prices or quantities) in most cases, economists have been slow to use the other kinds of information that are available. The paucity of theoretical literature may be a casualty of the slight empirical research, but it is still a bit of a puzzle. Illegal market activities, conducted against the authority of the state, pose interesting conceptual issues. Similarly, one might be curious about the contest between the impersonal forces of the market and the forces of the visible hand of violence.

Industrial organization, the subdiscipline in which studies of illegal markets would fall, is a rich field (4). It rightly concludes from a long list of industry-specific studies (on automobiles, airlines, electricity, and even cement and concrete plants) that each has important distinctive characteristics. There are few generalizable results because specific aspects of the production technology, demand function, and context determine the principal outcomes (5). Hence, understanding an industry, and developing policy toward it, requires supplementing general economic principles with industry-specific insights.

Thus, it is hardly surprising that a meta lesson from the papers in this Special Feature is that illegal markets present a similar opportunity. In addition to idiosyncrasies comparable to those of each legal market or industry, illegality itself introduces other important distinctive features, in particular the intensity, targeting, and integrity of enforcement. Thus, there is a need for focused study both of individual illegal markets, and also of illegal markets as a class.

While most market participants are “economic agents”–in the sense of acting in their own self-interest–the study of illegal markets and supply chains is not just a niche within economics for two reasons. First, illegal markets have distinctive characteristics that matter, such as the inability to enforce contracts in civil courts. Second, the wisdom and tools of other disciplines can be especially relevant. For example, it is not possible to understand illegal firearm markets without drawing on criminology, and impossible to regulate gun access effectively without accounting for the dynamic adaptation of illegal markets to those regulations. Illegality has profound effects on how enterprises are organized and who chooses to participate in these markets, and illegal markets have profound effects on the success–and failure–of efforts to ban certain types of goods, services, or production methods.

A second premise of this Special Feature is that illegal markets as a class cannot be understood by studying just one member of that class. Only by comparing and contrasting multiple illegal markets can one begin to identify their essence. Consider, for example, the proposition that illegal markets breed violence. A small number of markets do indeed generate high levels of violence, at least in some places and at some times. Drug markets in Latin America stand out in that respect (6, 7). However, even for drugs, cryptomarkets seem to produce very low rates of violence (8), and markets for human smuggling, counterfeit goods, or even loansharking are rarely described as having high levels of competitive or transactional violence (9, 10). Customers of human smuggling/trafficking organizations suffer high rates of death, but that mostly reflects accidents not homicides given the inherent risk of clandestine voyages across seas. What might explain the variation in violence: characteristics of the product, the intensity or nature of enforcement, characteristics of the buyers or sellers? Probably all of these and yet other environmental factors (such as the availability of guns and income inequality) have a role. Moreover, the current level of violence in an illegal market may also influence who enters it and, hence, how violent it is in the future.

Furthermore, illegal markets’ impact and importance can exceed that suggested by revenue alone. The loss to humanity when endangered species are poached and sold can far exceed the revenue those sales generate because of a range of market failures (11) and people’s willingness to pay for nonuse value (12). When terrorists or revolutionaries finance violent activity with the proceeds of illegal sales, the consequences can be great (13, 14). The FARC, a major left-wing terrorist group, was able to extend its bloody battle with the Colombian government because of its taxation of coca farmers and cocaine exporters (15).

There are three main goals for this Special Feature. One is to describe various illegal markets with a special focus on their structure and how they operate. Perhaps for the first time, this volume gathers in one place papers by some of the world’s leading experts on nine different illegal markets, all tasked with describing “their” market via a common analytic lens. The second goal is to synthesize the main findings from these articles, focusing on analytic commonalities and implications for policy. The final goal is to raise questions that may motivate other researchers to work on these topics, possibly spurring conversations about whether illegal markets should become a more prominent field of scientific inquiry.

The remainder of this introduction is organized as follows. The next section describes the structure of the nine individual chapters and the overall volume. Section 3 highlights some of the heterogeneity across and within the nine markets, while Section 4 specifically focuses on the role that organized crime groups play in these markets. The final section briefly mentions some of the important markets that are omitted from this volume.

## 2. Structure of This Volume and the Individual Papers

As far as we know, this is the first attempt to describe multiple illegal markets using a common framework. But to be clear, we are not claiming there has not been extensive research about these markets and their participants. To the contrary, some of these markets are central to fields of research that have their own peer-reviewed journals and academic organizations; however, most of that research does not focus on the markets as markets, or the participants as economic agents. A typical study might document the volume of illegal activity (i.e., the market clearing quantity), who is harmed by that market activity, and/or the sociological context from which the market participants are drawn, but not view the markets or the supply chains as the analytical unit being studied. Likewise, for most of these markets, the focus on the economic motivations driving the structure and nature of the enterprises involved in the supply chain has been limited, at best.

The common framework here ensures that each of the market-specific articles address seven areas: 1) Overview of the market; 2) Origins of prohibition; 3) Enforcement and policy; 4) Scale of the market and harms; 5) Structure and nature of enterprises in the supply chain; 6) Market developments; and 7) Ideas for future research. As editors, we gave authors flexibility, so the order of those seven topics may differ across chapters and authors sometimes include additional sections about “their” markets.

The final two chapters synthesize insights about these markets. One focuses on the “basic science,” asking what fundamentally these different markets have in common (16). The other takes an “applied science” perspective, asking what are the implications for policy (17).

The chapters are ordered to discuss markets for illegal services first and then illegal goods.

The services chapters are ordered by frequency of purchase, from aspiring immigrants who hope to purchase human smuggling assistance just once, to criminals buying money laundering services repeatedly, to commercial sex, which has high-frequency as well as occasional customers. Within goods, the ordering is from thinner markets for less frequently purchased durable goods to thicker markets for frequently purchased nondurables such as drugs. An advantage of this ordering is putting the best-studied illegal markets—those for illegal drugs—at the end, to reduce the risk, observations about drug markets get overgeneralized.

Both as a teaser and to drive home the point made above, that illegal markets as a class cannot be understood by studying just one member of that class, we note for each a way that the market differs from drug markets–which are perhaps the most discussed illegal market.

Human smuggling: Most illegal drugs are consumed by people who purchase daily or near-daily and so often buy thousands of times over a multiyear “career” of use. By contrast, people contracting with human smugglers for assistance crossing international borders hope to only purchase that service once in their lifetimes.

Money laundering: Most illegal drugs are consumed by those who spend a sizable share of their disposable income purchasing the drug and impoverishment is common among the heaviest users. By definition, only people with large amounts of (dirty) money have any reason to hire someone to launder their funds. More generally, the welfare of customers is simply not a concern when it comes to making policies that affect money laundering markets.

Commercial sex: Most illegal drugs are commodities supplied by multilayered transnational supply chains that may hold a year or more’s worth of consumption in inventory at any time. By contrast, traditional in-person commercial sex is a personal service that must be produced locally and simultaneously with consumption.

Firearms: Firearms are a durable good that is purchased infrequently, making the market for illegal firearm transactions so thin as to raise the question of whether it is actually a well-defined market with going market prices. By contrast, there are literally billions of illegal drug sales every year in the United States alone. Also, drugs generally flow from the global south to the global north; more illegal guns cross the US–Mexican border from north to south than from south to north.

Counterfeit goods: Heroin is illegal because heroin is dangerous, and powerful opioids can kill no matter who manufactures them. By contrast, a counterfeit Chanel purse is not dangerous. Indeed, the purse itself does not even become illegal until someone other than Chanel attaches to it the distinctive Chanel mark (the outward facing, intersecting double C’s). Yet in terms of revenue, counterfeit goods are the only illegal market to rival drug markets in size.

Wildlife: Drugs can harm consumers, e.g., through overdose and addiction. Illegally trafficked wildlife products rarely do; no musician has died from playing a piano with ivory keys. Rather, the societal harms motivating the prohibition of IWT trafficking pertain to production not consumption.

Illegal, Unregulated, and Unreported (IUU) fishing: Drug law enforcement is a government activity pursued by police, prosecutors, and prison systems. To be sure, Coast Guards and other enforcement agencies combat IUU fishing, but nonprofits also play a prominent role in efforts to detect and thwart IUU fishing. Also, prescription opioids get diverted from legal supply chains into illegal street markets, but IUU fish get diverted from illegal production into legitimate supply chains that supply fish to (legal) restaurants and grocery stores.

Tobacco products: Traditional illegal tobacco markets sold products that were comparable to tobacco products sold legally; the crime was evading taxes, not selling something that was intrinsically illegal. Now, though, there are outright bans in Australia and elsewhere on e-cigarettes, a new nicotine product that did not exist 30 years ago. By contrast, many major illegal drug markets sell products that have existed for a century or more.

Drugs: During Prohibition, alcohol often sold for around $6 per gallon, which is about $0.04 per gram in today’s dollars. By contrast, wholesale prices for heroin, cocaine, and meth are roughly $20,000 per kilogram, or $20 per gram. That the value to weight ratio for illegal drugs is approximately 500 times that of alcohol during Prohibition greatly affects smuggling practices and enforcement opportunities.

## 3. Heterogeneity across and within Illegal Markets

Perhaps the most striking thing one encounters when reading the papers in this Special Feature is how diverse illegal markets are. While the synthesis chapters provide rigorous comparisons and thematic analyses, here we offer a few insights about the heterogeneity across and within them.

**Fig. 1.**
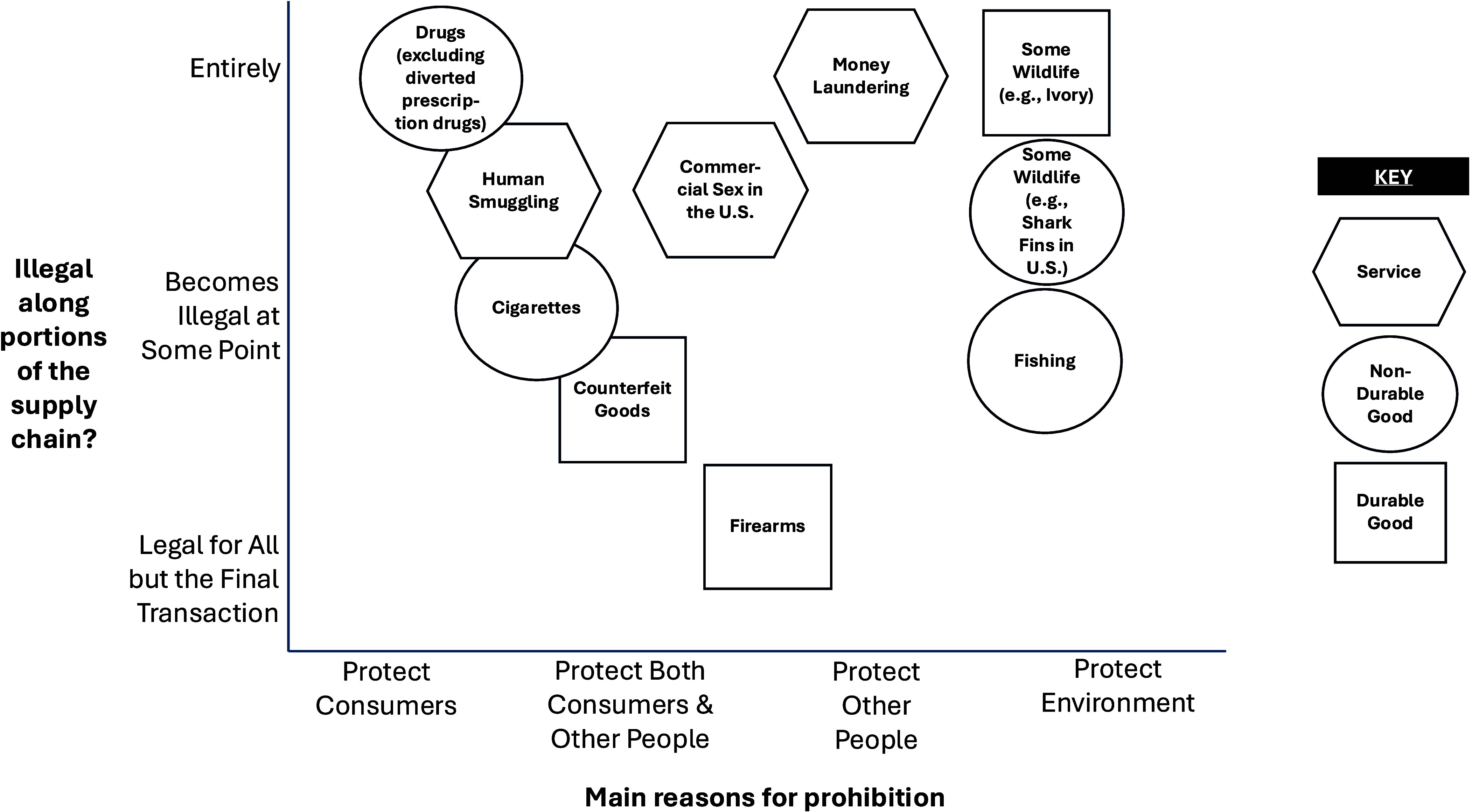
One framework for comparing illegal markets.

Heterogeneity across markets. There are multiple ways to classify these markets. Fig. 1 presents a framework focused on three dimensions: The shapes denote the type of good or service, the Y-axis captures the extent to which the product is illegal all along the supply chain, and the X-axis reflects who or what was intended to be protected by the prohibition giving rise to the illegal market.

Hexagons denote services. For example, Ferwerda describes money laundering as a service that “repairs” money that is “damaged” by making money obtained from illegal activities look legal (18). That analytical perspective is more useful for understanding money laundering markets than are the legal definitions which often define merely possessing or spending any money derived from criminal activity as money laundering.

The other shapes in Fig. 1 represent durable (square) and nondurable (circle) goods. Most markets are one or the other, but as Midgette and Gore note, some wildlife products are durable (e.g., ivory) while others are nondurable (e.g., shark fins used in soups) (19), so there are two symbols for wildlife trafficking. Other noteworthy variations within markets are discussed in the next subsection.

The Y-axis shows that some articles of illegal commerce are illegal throughout the supply chain (e.g., heroin, elephant ivory), but for others the illegality stems only from who, when, or how the transaction occurs. For example, illegal firearms are ranked the lowest on the Y-axis because, as Cook and Smart note, the gun itself might be a perfectly legal object, if only it were being sold to someone else (20). Many other products are intermediate as they are legal in a portion of the supply chain but illegal at the other end (e.g., IUU fish and also tobacco that is legally produced but diverted at wholesale without tax stamps).

The X-axis focuses on variation in the nature of the harms associated with the banned activities, or the motivation for their prohibition. Drug prohibitions have many motivations, but prominent among them is protecting consumers from harming themselves via addiction or overdose. The same holds for bans on e-cigarettes. Bans on counterfeit goods can protect the consumer (e.g., for low quality counterfeit medicines), or—in the case of designer handbags—the profits of the trademark holders (among other considerations). Bans on IUU fishing aim to protect the environment and the property rights of those who own the fishery. Enforcement against money laundering is not directly motivated by any harms of money laundering per se; those are rather modest (21). Rather, the goal is to impede the crimes that generate the funds, and to prevent criminals from enjoying the fruits of their nonmoney laundering crimes

One could add other dimensions to Fig. 1, such as whether the final customers are knowingly engaging in criminal activity. For those buying drugs or human smuggling services, the answer is clearly yes. Illegal fishing can sometimes be different. As LeBillon, Sumaila, and Ladefoged describe, those consuming fish at home or in restaurants may have no idea whether their meal was harvested legally or not (22). There are multiple points in the supply chain where “illegal fish” get comingled with fish caught legally and so enter the legitimate market. Kenkel and Phillips show that illegal cigarettes are in between, as some people know they are purchasing an illegal or tax-evading carton or pack, but not everyone does (23).

Illegal markets rapidly harness new technologies, sometimes transforming the markets in fundamental ways. Even though commercial sex has been dubbed “the world’s oldest profession”, Shah (24) notes that the last decade has brought radical changes. OnlyFans and other online marketplaces have upended those markets. What was once inherently a locally supplied service, now spans boundaries as readily as the internet, and creates winner-take-all potential that simply did not exist before (25).

Likewise, democratized international payment systems facilitate human smuggling by sparing “customers” from having to carry cash when they are vulnerable to predation. Direct B-to-C internet sales, globalization of commerce, and the proliferation of contract manufacturing, as well as technological innovations such as 3-D printing, have fueled an explosion in counterfeit goods markets. Cryptocurrencies create new opportunities for money laundering.

Policy changes have transformed other markets. Some have been eliminated or at least greatly challenged by competition from newly legal markets, including sports betting in the United States and cannabis markets in Canada, Uruguay, and roughly half of US states. New commercial legal markets have spawned a proliferation of new products for both gambling (proposition bets, in-play betting, quarter and half lines, etc.) and cannabis (vapes, concentrates, infused prerolls, tinctures, lotions, etc.). Meanwhile, new prohibitions are enacted, particularly those motivated by environmental protection, and entirely new goods and services appear (e-cigarettes, online commercial sex, phony carbon offset certificates) creating new markets with variable legal status in the sense that what is legal varies by country, consumer status, and/or intended use (26).

Heterogeneity within market categories. Saying this Special Feature has nine papers on nine markets is a bit misleading. Each is really a category, not a single market, and there can be great variation within each category. Kilmer highlights the varied supply chains for different illegal drugs (27). The market for cocaine, with a logistics chain stretching from rural Colombia to the streets of Munich, presents policy and analytic challenges distinct from those of the US psilocybin mushroom market, which is almost entirely domestic and with few steps from grower or forager to user. The psilocybin logistics chain might seem more vulnerable, having no international borders to hide behind, but it is also impervious to the vagaries of growing conditions and does not generate large quantities of cash that require laundering.

Kennedy shows how the market for counterfeit, high-fashion handbags is completely distinct from that for counterfeit medicines (28). The former involves primarily intellectual property theft from large enterprises, with the benefits going to a wide range of socioeconomic classes. Counterfeit pharmaceuticals, often manufactured in China or India for sale in low-income African countries, can provide real benefits to populations whose access to legitimate medicines is limited by both regulation and income, but also real costs when the counterfeit medicines are contaminated or fake. Also, the intellectual property being stolen, often the result of large investments in research, is arguably more socially valuable than that of high fashion manufacturers.

Likewise, laundering of drug traffickers’ weekly cash earnings requires very different services from those provided to high-level kleptocrats who at irregular intervals receive large payments delivered digitally into bank accounts through complex chains of transactions (29). Typical low-level wholesale drug dealers may “self-launder” by making cash purchases or use unskilled “smurfs” to deposit cash a few thousand dollars at a time. The laundering of funds looted by kleptocrats is often conducted by lawyers, accountants, and other highly sophisticated professionals, who know how to create lengthy chains of transactions involving Luxemburg Trusts, Cayman Island shell companies, and Panamanian lawyers (30). Regulations that work against the simple cash concealing schemes may do little to affect the more complex version, and vice versa.

Sometimes what seems like great variation reduces to common core business operating principles. Human smuggling may require boats to cross the Mediterranean or English Channel vs. desert treks across the Mexican-US border, but that is probably a mere detail. The analytic issues may be similar regardless of the means of conveyance.

One implication of this variation is that the field of illegal market studies will benefit from more granular studies. The unit of observation may have to be a specific kind of product in an individual country at a specific time. Deciding how to categorize the product for these purposes will be one of the challenges.

## 4. Role of Organized Crime in Illegal Markets

The specter of organized crime hangs over all these markets. Enforcement agencies, politicians, and the media routinely claim that all of these markets are in the grip of, or generating fortunes for, organized crime.^*^ In fact, the evidence for this is mixed; the role of organized crime varies by the nature of the market and across countries. The same market in country A may be independent of organized crime, but in country B be largely operated by, or extorted by, organized crime.

Serious economic analysis of organized crime must attend to definition considerations (31). Many statutory definitions are driven by (valid) prosecutorial considerations, but they can trivialize the phenomenon. For the United Nations, an organized crime group is any “group of three or more persons, existing for a period of time, acting in concert with the aim of committing at least one crime punishable by at least four years” incarceration in order to obtain, directly or indirectly, a financial or other material benefit’ (32). Under that definition almost all market enterprises involve organized crime. However, as illuminated by a large literature, distinctive harms arise from the more elaborate and established organizations that are often loosely characterized as “mafias”. Those criminal organizations pose distinctive threats to the societies in which they flourish (33, 34); for example, their corrupting of high-level officials and sometimes creation of their own political base undermine the legitimacy of governments.

A newer literature emphasizes distinctions among the principal goals of organized crime groups, including the distinction between production/trade groups and governance groups (35). “In illegal markets, criminal governance often involves protection against competition, enforcement of cartel agreements, dispute resolution, labor racketeering, intimidation of lawful right-holders, protection against theft and police harassment, informal credit and debt recovery, and defence against extortionists” (36).

Many illegal markets have been important sources of money and/or power to mafias. Drug markets occupy a particularly prominent position in that narrative. ‘Ndrangheta, a mafia originating in Calabria, is thought to be a dominant figure in European cocaine trafficking (37). Brazil’s most important organized crime group, the Sao Paolo PCC, is thought to derive more revenue from cocaine dealing than from any other criminal activity (38). In nations without a small number of powerful mafias, many drug markets seem to involve participation by more local organized crime groups (35).

In markets dominated by myriad small groups, the individual groups can come and go. Indeed, a great frustration of law enforcement is that taking down organizations, even prominent ones, may not interrupt supply. But the character and conduct of supply chains are hardly immutable. Over a 50-year period, US drug markets have seen great changes in who are the most prominent groups; the US Mafia in the 1970 s, Colombian groups between about 1980 and 2000 and, since then, Mexican groups (39). Furthermore, whereas for the US Mafia, drugs were one line of business among many, since 1980 the drug trafficking groups within US borders have been more specialized enterprises; the Colombian and Mexican groups are simply drug distribution businesses in the United States, with no governance structures visible.

For most other illegal markets, the relationship with organized crime is much weaker. Though early twentieth century history is replete with the Mafia running brothels (40), it now seems that there is little contemporary involvement in either the in-person or growing internet versions of commercial sex. Money laundering services are more often purchased by mafias rather than provided by them (41). Human smuggling, even to Italy with its three prominent mafias (Sicilian Mafia, ‘Ndrangheta, Camorra), has not seen any involvement of them (42).

## 5. Other Illegal Markets

The nine markets included in this volume are among the most important, both in terms of the illegal revenues and the harms they produce; however, we could not include every illegal market. This section briefly reviews some important markets that we could not cover, identifying what the existing literature suggests might be distinctive about each.

Gambling. Gambling comes in many forms, and its legality varies over time and place. In the United States in 1960 only horse race betting and bingo, a small-scale form of lottery, were allowed and in only some states. Now the most popular forms of gambling (lottery, casino, sports betting) are legal in many states, and online.^†^ As a result, the illegal gambling market, described in the 1960s as the principal source of revenue for the American Mafia (43), is now much shrunk. Legal gambling opportunities have increased in many other countries as well, but some large nations still have substantial illegal betting markets.

In Germany the issue of illegal gambling is prominent enough that the coalition coming into power following February 2025 elections included positions on reducing it in their platform (https://igamingbusiness.com/offshore-gaming/germany-new-government-illegal-gambling/). In India, where states rarely allow more than lottery and horse race betting, it is estimated that most gambling is in violation of state laws (44). In China, whose government has banned all forms of gambling except 1) state lotteries (which it claims is not gambling!) and 2) casinos in Macau, there are believed to be many illegal casinos (45).

Loansharking. Loansharking is the lending of money with an explicit or implicit threat of violence or aggressive hectoring of the borrower by the lender, at very high rates of interest. This exists in every society but is prominent in only a few. A recent study in Singapore found that it was common among Chinese residents with gambling or drinking problems (46). As with so many other markets, the internet has changed much: “To enforce loan repayment, lenders have developed a new strategy: relational repression, which is the use of cyberviolence and the threat of revealing damaging information to clients’ social contacts. This puts enormous pressure on clients and their families to make payments. The use of relational repression reduces the need to resort to physical violence and bribe police officers.” (47)

Other markets. There are many less well-known illegal markets, including from illegal mining which in a way parallels illegal harvesting of fish and wildlife. For example, some countries have large illegal markets in sand, used principally in construction (48). Legal mining requires permits and the demand for sand in China, with its longstanding and extraordinary construction boom, far exceeds legal supply. As the Economists noted in 2022, “No country is hungrier for [sand] than China, where the damage caused by rampant illegal mining has been enormous” (49). Antiquities, whose discovery and trade are subject to strict regulation, are often sold in violation of those regulations. This can generate important streams of revenue for some nations in conflict, as in the Middle East or Southeast Asia.

The set of major illegal markets varies over time. While we have no prediction as to which will emerge and which will close in the next 25 years, we are confident that 1) there will be change and 2) there will continue to be a need to rigorously study the structure and nature of these markets.

## Data Availability

There are no data underlying this work.

## Footnotes

^*^Illegal cigarettes: “For decades, cigarette smuggling has been a sizeable and dependable revenue stream for organized crime.” https://2009-2017.state.gov/documents/organization/250513.pdf. Human smuggling: “The fact that migrant smuggling networks are closely linked to other forms of serious and organised crime including terrorism, trafficking in human beings and money laundering increases this urgency.” https://home-affairs.ec.europa.eu/policies/migration-and-asylum/irregular-migration-and-return/migrant-smuggling_en. International Wildlife Trafficking: “With the black market for illegal wildlife products worth up to USD 20 billion per year, poaching and the illegal wildlife trade has become a major area of activity for organized crime groups…”https://www.interpol.int/en/News-and-Events/News/2023/Illegal-wildlife-trade-has-become-one-of-the-world-s-largest-criminal-activities.

^†^For a 2024 listing of large countries and whether they permit internet gambling (iGaming) see https://www.scaleo.io/blog/igaming-regulations-around-the-world-what-you-must-know/.

## References

[1] Statista, Worldwide consumer electronics market revenue. https://www.statista.com/forecasts/1286653/worldwide-consumer-electronics-market-revenue (Accessed 14 May 2025).

[2] A. E. Roth, Marketplaces, markets, and market design. Am. Econ. Rev. **108**, 1609–1658 (2018). 30091861

[3] G. S. Becker, K. M. Murphy, M. Grossman, The market for illegal goods: The case of drugs. J. Polit. Econ. **114**, 38–60 (2006).

[4] T. Tirole, The Theory of Industrial Organization (MIT Press, 1988).

[5] L. Einav, J. Levin, Empirical industrial organization: A progress report. J. Econ. Perspect. **24**, 145–162 (2010).

[6] A. Durán-Martínez, To kill and tell? State power, criminal competition, and drug violence. J. Confl. Resolut. **59**, 1377–1402 (2015).

[7] P. Miraglia, Drugs and drug trafficking in Brazil: Trends and policies. Center for 21st Century Security and Intelligence Latin America Initiative (2015).

[8] M. J. Barratt, J. A. Ferris, A. R. Winstock, Safer scoring? Cryptomarkets, social supply and drug market violence. Int. J. Drug Policy **35**, 24–31 (2016). 27241015 10.1016/j.drugpo.2016.04.019

[9] P. Campana, Human smuggling: Structure and mechanisms. Crime Justice **49**, 471–519 (2020).

[10] K. Lang, K. Leong, H. Li, H. Xu, Borrowing in an illegal market: Contracting with loan sharks. Rev. Econ. Stat. **107**, 269–278 (2025).

[11] I. M. Fraser, D. L. Roberts, M. Brock, The economics of species extinction: An economist’s viewpoint. Camb. Prisms Extinct. **1**, e20 (2023). 10.1017/ext.2023.18 PMC11895705 40078692

[12] L. Richardson, J. Loomis, The total economic value of threatened, endangered and rare species: An updated meta-analysis. Ecol. Econ. **68**, 1535–1548 (2009).

[13] C. Musu, “Cultural heritage protection, illicit antiquities, and the international fight against terrorism financing” in The Safety and Security of Cultural Heritage in Zones of War or Instability, B. Romiti, A. Folliero, Eds. (IOS Press, 2021), pp. 15–31.

[14] A. Hiropoulos , “Cigarette smuggling and terrorism financing: A script approach” in Cognition and Crime, B. Leclerc, R. Wortley, Eds. (Routledge, 2013), pp. 186–208.

[15] F. E. Thoumi, Illegal Drugs, Economy, and Society in the Andes (Woodrow Wilson Center Press, 2003).

[16] J. P. Caulkins, A budding science of illegal markets. Proc. Natl. Acad. Sci. U.S.A. **123**, e2511780123 (2026). 42574616 10.1073/pnas.2511780123 PMC13486562

[17] L. Paoli, P. Reuter, The policy construction of illegal markets: Exploring variation, success, failure and unintended consequences. Proc. Natl. Acad. Sci. U.S.A. **123**, e2515300123 (2026). 42574610 10.1073/pnas.2515300123 PMC13486593

[18] J. Ferwerda, The money laundering market: An economic analysis. Proc. Natl. Acad. Sci. U.S.A. **123**, e2512080123 (2026). 42574621 10.1073/pnas.2512080123 PMC13486520

[19] G. Midgette, M. L. Gore, How illegal wildlife trade adheres to and defies conventional market behavior. Proc. Natl. Acad. Sci. U.S.A. **123**, e2525056123 (2026). 42574631 10.1073/pnas.2525056123 PMC13486546

[20] P. Cook, R. Smart, Illegal firearms markets: Transaction patterns, evolving sources, and importance in criminal misuse. Proc. Natl. Acad. Sci. U.S.A. **122**, e2509754122 (2026). 10.1073/pnas.2509754122 PMC13486543 42574615

[21] J. Ferwerda, “The effects of money laundering” in Research Handbook on Money Laundering, B. Unger, D. van der Linde, Eds. (Edward Elgar Publishing, 2013), pp. 35–46.

[22] P. Le Billon, Z. Ladefoged, U.R., Sumaila, Casting light on the workings of illegal fishing markets. Proc. Natl. Acad. Sci. U.S.A. **123**, e2512081123 (2026). 42574605 10.1073/pnas.2512081123 PMC13486526

[23] D. Kenkel, G. Philips, Illegal markets for cigarettes and e-cigarettes. Proc. Natl. Acad. Sci. U.S.A. **122**, e2512084122 (2026). 10.1073/pnas.2512084122 PMC13486574 42574627

[24] M. Shah, The illegal market for sex in the United States: Insights from economic research. Proc. Natl. Acad. Sci. U.S.A. **122**, e2512375122 (2026). 10.1073/pnas.2512375122 PMC13486583 42574632

[25] R. H. Frank, P. J. Cook, Winner-take-all markets. Stud. Microecon. **1**, 131–154 (2013).

[26] P. Lord, P. Paoli, Regulating emerging illegal markets. (forthcoming).

[27] B. Kilmer, Stability and turbulence in illegal drug markets. Proc. Natl. Acad. Sci. U.S.A. **123**, e2512078123 (2026). 42574612 10.1073/pnas.2512078123 PMC13486525

[28] J. Kennedy, A descriptive analysis for the markets for counterfeit products. Proc. Natl. Acad. Sci. U.S.A. **123**, e2522322123 (2026). 42574613 10.1073/pnas.2522322123 PMC13486533

[29] M. Riccardi, P. Reuter, The varieties of money laundering and the determinants of offender choices. Eur. J. Crim. Policy Res. **30**, 333–358 (2024).

[30] J. Costa, The nexus between corruption and money laundering: Deconstructing the Toledo-Odebrecht network in Peru. Trends Organ. Crime **27**, 342-363 (2022).

[31] P. Paoli, The concept of organized crime: Theoretical and empirical implications. Trends Organ. Crime **23**, 3–17 (2020).

[32] United Nations, United Nations convention against transnational organized crime and the protocols thereto. https://www.unodc.org/documents/treaties/UNTOC/Publications/TOC%20Convention/TOCebook-e.pdf (Accessed 14 May 2025).

[33] D. Gambetta, The Sicilian Mafia: The Business of Private Protection (Harvard University Press, 1993).

[34] F. Varese, The Russian Mafia: Private Protection in a New Market Economy (Oxford University Press, 2001).

[35] P. Campana, F. Varese, Organized crime in the United Kingdom: Illegal governance of markets and communities. Br. J. Criminol. **58**, 1381–1400 (2018).

[36] P. Campana , Criminal governance in a large European city: The case of gangs in London. Eur. J. Criminol. **22**, 877–900 (2025). 41114276 10.1177/14773708251315581 PMC12530685

[37] F. Calderoni, The structure of drug trafficking mafias: The ‘Ndrangheta and cocaine. Crime Law Soc. Change **58**, 321–349 (2012).

[38] B. Lessing, G. D. Willis, Legitimacy in criminal governance: Managing a drug empire from behind bars. Am. Polit. Sci. Rev. **113**, 584–606 (2019).

[39] P. Reuter, “Drug markets and organized crime” in *The Oxford Handbook of Organized Crime*, L. Paoli, Ed. (Oxford University Press, 2014), pp. 359–381.

[40] S. Raab, Five Families: The Rise, Decline, and Resurgence of America’s Most Powerful Mafia Empires (Macmillan, 2016).

[41] A. Woolner, Washed in Gold: The Story Behind the Biggest Money Laundering Investigation in U.S. History (History Simon and Schuster, 1994).

[42] P. Campana, The market for human smuggling: Structure, mechanisms and paradoxes. Proc. Natl. Acad. Sci. U.S.A. **122**, e2511019122 (2026). 10.1073/pnas.2511019122 PMC13486519 42574635

[43] D. Cressey, Theft of the Nation: The Structure and Operations of Organized Crime in America (Harper & Row, 1969).

[44] S. George, R. Velleman, A. Nadkarni, Gambling in India: Past, present and future. Asian J. Psychiatr. **26**, 39–43 (2017). 28483088 10.1016/j.ajp.2017.01.018

[45] F. Master, Chinese state media target Macau’s Suncity in online gambling report. Reuters (2019), https://www.straitstimes.com/asia/east-asia/chinese-state-media-target-macaus-suncity-in-online-gambling-report.

[46] K. Lang , Borrowing in an illegal market: Contracting with loan sharks. Rev. Econ. Stat. **107**, 269–278 (2025).

[47] P. Wang, M. Su, J. Wang, Organized crime in cyberspace: How traditional organized criminal groups exploit the online peer-to-peer lending market in China. Br. J. Criminol. **61**, 303–324 (2021).

[48] A. Rege, Not biting the dust: Using a tripartite model of organized crime to examine India’s Sand Mafia. Int. J. Comp. Appl. Crim. Justice **40**, 101–121 (2016).

[49] Economist, China’s war over sand. https://www.economist.com/china/2022/06/14/chinas-war-over-sand (Accessed 20 May 2026).

